# Perceived Impact of Healthcare Relationships and Interactions on Parental Experiences of Prenatal Diagnosis and Termination of Pregnancy for Foetal Anomaly on the Island of Ireland

**DOI:** 10.1111/hex.70068

**Published:** 2024-10-20

**Authors:** Suzanne Heaney, Mark Tomlinson, Áine Aventin

**Affiliations:** ^1^ School of Nursing & Midwifery Queen's University Belfast Belfast UK; ^2^ Institute of Life Course Health Research Stellenbosch University Cape Town South Africa

**Keywords:** health and well‐being, healthcare relationships, PPIE, pregnancy loss, TFMR, TOPFA

## Abstract

**Objective:**

The aim of this study was to explore parents' experiences of their relationships and interactions with healthcare professionals (HCPs) during care related to prenatal diagnosis and termination of pregnancy for foetal anomaly (TOPFA).

**Methods:**

A qualitative approach was used. Participants included 33 parents (23 women and 10 men) from Northern Ireland (*n* = 11) and Ireland (*n* = 22) who had a TOPFA. Data collection methods included semi‐structured interviews and written narrative accounts. Data were analysed using thematic analysis.

**Results:**

Findings confirmed that TOPFA was a traumatic, life‐altering experience for parents, impacting their health and well‐being. The actions, behaviours and words of HCPs impacted how parents perceived and interpreted their healthcare experiences and their access to services and supports. In relation to this, five themes are presented: (1) the importance of compassionate and non‐judgemental care, (2) the value of effective information and communication, (3) the desire for compassionate care for baby and facilitation of memory making, (4) the need for continuity of care and (5) parents' experiences of healthcare relationships during times of legislative change.

**Conclusion:**

This research reveals the important role HCPs play in helping parents cope with prenatal testing and TOPFA. Parents who had a positive relationship with an HCP, in which information was communicated effectively and compassionate and non‐judgmental care was provided, felt more supported and more able to accept and adapt to their loss.

**Patient and Public Contribution:**

An advisory group composed of parents who had experienced TOPFA and HCPs with experience in caring for such families were involved in the study from the outset, contributing to the design and development of data collection materials, interpretation of the findings and design of dissemination materials.

## 1 Introduction

The term ‘congenital anomaly’ includes a broad range of structural, chromosomal, genetic and biochemical abnormalities present at birth, which often have significant consequences for the affected baby and their family [1]. Improved technology and advances in clinical testing have resulted in increased detection rates of congenital anomalies during the antenatal period [2, 3, 4, 5]. However, detection rates vary among European countries, and reported figures range between 30% and 80% according to data obtained from European registers [6, 7]. The number of pregnancies globally associated with congenital foetal anomalies annually is approximately 3% [8, 9, 10].

Although detection rates have improved, there are limited in utero treatments available for major anomalies [11, 12, 13, 14], leaving parents with limited options following diagnosis. They can continue with the pregnancy, or they can request a termination of pregnancy (TOP) in one of the 113 (57%) countries and territories around the world where it is legal to do so [3, 15, 16]. Termination of pregnancy for foetal anomaly (TOPFA) is the term used to describe the deliberate ending of a pregnancy due to a prenatally diagnosed foetal anomaly [17]. Countries vary with legal definitions and provisions about the nature and severity of anomalies to be eligible for a TOPFA. As such, access and availability can vary significantly between and within countries. Qualifying terms such as ‘non‐viable’, ‘fatal’ or ‘serious’ lack clear medical definitions, which can lead to a lack of clarity as to whether a TOPFA is legally permissible or not, resulting in variations in access and availability [18].

Laws governing access to abortion care, including TOPFA in Ireland (IE) and Northern Ireland (NI) were reformed in both jurisdictions in 2018 and 2019, respectively. Before this, access to TOP in both regions was only available in extremely limited circumstances, and women had to travel to other jurisdictions (usually mainland United Kingdom) to access care [19, 20]. In IE, the Health (Regulation of Termination of Pregnancy) Act 2018 legalised abortion, which has been available free of charge since January 2019 [21]. Regarding TOPFA, the legal guidelines state that for a woman to be eligible to access a TOPFA, two medical practitioners must be of the agreed opinion, in good faith, that the baby will die during pregnancy, labour or within 28 days of birth [22]. This clause means some women who receive a foetal anomaly diagnosis in IE still need to travel to access TOPFA care. Abortion in NI was decriminalised in October 2019 following the introduction by the UK government of the Northern Ireland Executive Formation Act 2019 [23]. The Abortion (Northern Ireland) Regulations provide a framework for lawful abortion services [24], including in cases of ‘severe fetal impairment or fatal fetal abnormality’ (Regulations, Section 7). Following a delay in the commissioning of abortion services in NI, the NI Office led commissioning from 2022, and full services were established in late 2023 [25, 26, 27, 28].

## 2 Rationale for Study

This study responds to a gap in the research evidence regarding the healthcare experiences and needs of parents experiencing TOPFA on the Island of Ireland. In this article, we explore the perceived impact of healthcare relationships and interactions on the healthcare experiences of parents to establish their healthcare needs and inform the design and delivery of newly established and commissioned services. This study is the first of its kind in NI and one of very few in IE.

## 3 Methods

The study is reported according to the Standards for Reporting Qualitative Research (SRQR) guidelines [29].

### 3.1 Qualitative Approach and Research Paradigm

A qualitative methodological design was adopted for this study [30]. An interpretivist approach facilitated the exploratory nature of this work, and contextualism allowed an understanding of individual perspectives within the context and conditions in which they experienced their TOPFA [31, 32].

### 3.2 Researcher Characteristics and Reflexivity

The first author conducted this study as part of her PhD research under the supervision of the other authors. She is a registered midwife, and the other authors are psychologists. The first author engaged in reflexive practice throughout the study, keeping a reflective journal and engaging in mentorship with supervisors, both of which involved reflection on progress, challenges encountered and decisions made.

### 3.3 Context

The research setting for this study was the island of Ireland (hereafter Ireland), which includes both Ireland (IE) and NI. IE covers approximately five‐sixths of the island with an estimated population of 5.1 million [33]. NI, which is part of the United Kingdom, covers one‐sixth of the island with a population of 1.88 million [34]. An estimated 63% of the population lives in an urban area in IE [35, 36] with 86% of the population in NI living in an urban area [37].

NI is one of the four constituent countries that comprise the United Kingdom. It has a devolved government and, as such, has different laws and arrangements for healthcare, including reproductive healthcare, to the rest of the United Kingdom. In NI, health and social care services are combined under what is known as Health and Social Care (HSC). Despite being created separately from the United Kingdom's NHS, NI's health service is considered part of the overall NHS and is free at the point of delivery. All women in NI are eligible for free maternity care provided by HSC Trusts. There are no private maternity hospitals in NI; therefore, all care during labour and birth is provided in HSC Trust facilities. However, some women choose to access private antenatal or postnatal care, usually in addition to NHS‐provided care.

The healthcare system in IE is unusual within Europe in not providing universal, equitable access to primary healthcare, with healthcare delivered through both public and private services. Approximately 43% of the population (mainly higher income groups) avail of private health insurance, which is mainly used to access private healthcare, supplied in both public and private hospitals [38]. All maternity services are provided free of charge in IE; however, both semi‐private and full‐private maternity care options are available.

### 3.4 Patient and Public Involvement

An advisory group (AG) composed of parents who had experienced TOPFA and healthcare professionals (HCPs) with experience in caring for such families were involved in the study from the outset, contributing to the design and development of data collection materials, interpretation of the findings and design of dissemination materials. AG members were identified via the authors' professional networks and included two women who had experienced or been affected by a diagnosis of a foetal anomaly and TOP, a registered mental health counsellor to advise on the mental health–related elements of the study and a representative from a charity, Antenatal Results and Choices (ARC), which supports parents and HCPs through antenatal screening and its consequences. None of the AG members took part in the research.

### 3.5 Participant Recruitment and Selection

**Figure 1:**
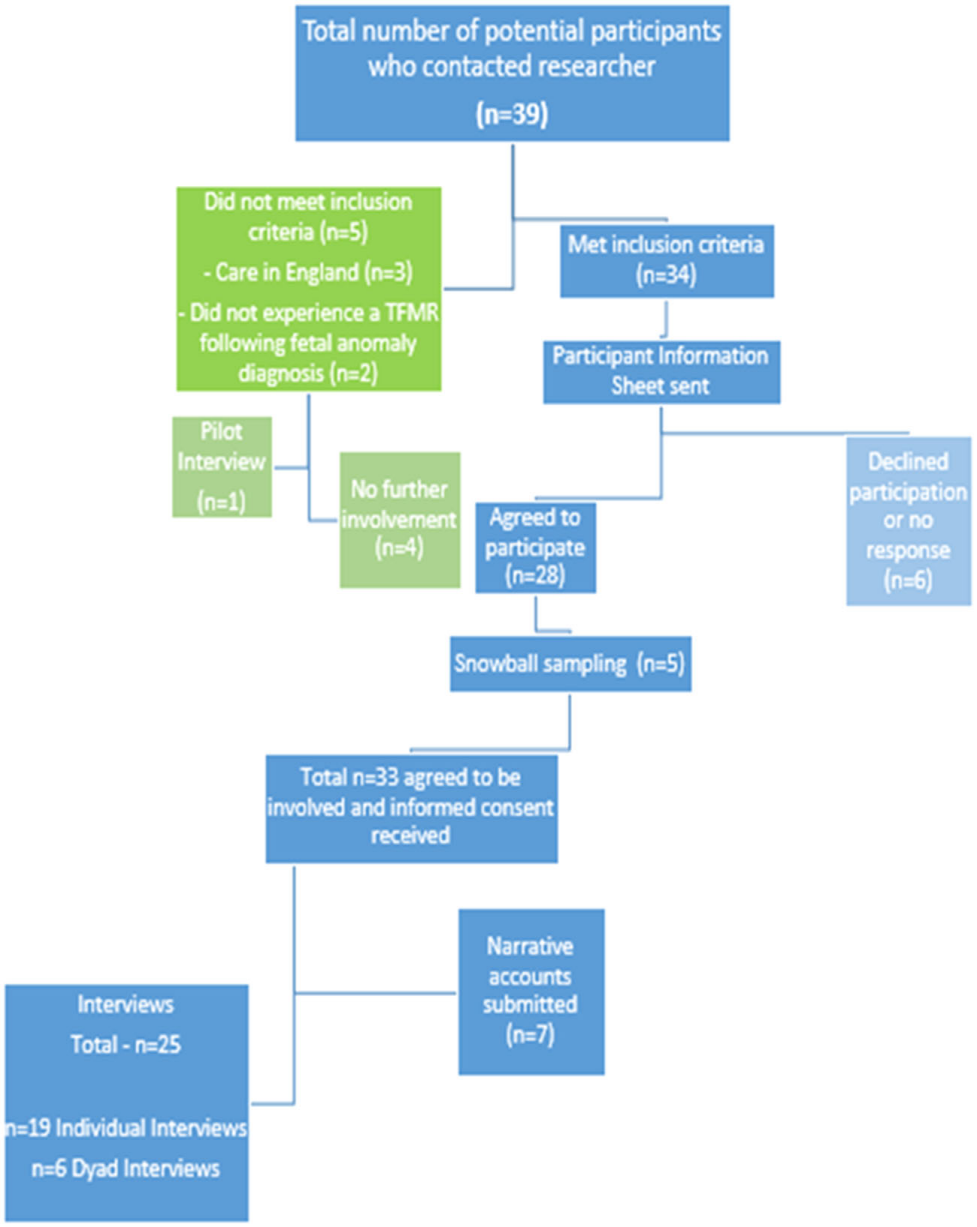
Flowchart of recruitment.

The AG consulted on recruitment procedures and content, helping to ensure the suitability and acceptability of the research. Participants were recruited through purposive and snowball sampling (Figure 1). An animation and recruitment poster were circulated on social media platforms through a range of forums, groups and social media such as Alliance for Choice, ARC, Informing Choices NI, Leanbh Mo Chroi, SANDS NI and TFMR Ireland. In the context of this study, purposive sampling refers to the recruitment of participants who met the inclusion criteria. Parents were eligible to take part if they were over 18, had a TOPFA in the past 10 years and, at the time of diagnosis, were living in IE or NI. All participants who contacted the researcher and met the inclusion criteria were invited to participate in the study.

### 3.6 Ethical Considerations

Informed written consent was obtained from each participant before the commencement of data collection. Participants also verbally confirmed consent before beginning an interview. Parents were advised of their right to withdraw from the study at any stage without reason. A Study Distress Protocol was designed to safeguard participants in case their participation resulted in emotional distress. All participants were signposted to support organisations.

Ethical approval was obtained from Queen's University Belfast, Faculty of Medicine, Health and Life Sciences Research Ethics Committee in May 2020 (Ref: MHLS 20_40).

### 3.7 Data Collection Methods and Instruments

Parents were offered the option to take part in a semi‐structured interview (individually or couple dyad) and/or to submit a written narrative account of their TOPFA experience. Interviews provided flexibility and freedom to the researcher to delve deeper into thoughts, feelings and areas that arose during an interview [39]. A narrative account was offered as an option to complement interview data or as an alternative if a participant was unable to attend an interview or preferred written medium. To encourage participation, participants were originally offered a choice of face‐to‐face, telephone or video call for their interview. However, due to COVID‐19 travel and social distancing restrictions, all except one interview were carried out via video call.

An interview guide (Supporting Information) was developed based on the research objectives and a systematic review of international literature on the topic [40], in consultation with the project AG. Interviews took an average of 90 min, were carried out by the first author, audio recorded and transcribed verbatim. Narrative accounts ranged from 1180 to 5594 words. All data were managed in the software system, NVivo 12 [41].

### 3.8 Data Analysis

The data from both interviews and narrative accounts were analysed by the first author when data collection was complete. The author used thematic template analysis following the seven‐step recursive process developed by King and Brooks [31]: (1) researcher immersion in data through critical reading of transcripts, interview field notes and reflections; (2) identification of a priori themes and inductive line‐by‐line preliminary coding; (3) initial codes clustered into meaningful groups; (4) after coding five transcripts and reviewing and clustering codes, an initial template was developed. This template was applied to five more transcripts; (5) following Step 4, codes were reviewed again, and the template further developed; (6) the template developed in Step 5 was then applied to the entire data set; (7) findings were written up for presentation.

### 3.9 Trustworthiness

To strengthen the credibility of the study, member checking [32] was offered to all participants after they completed their interview. Of the 33 parent participants, 22 wished to receive a member check. The researcher summarised the participant's contribution in a 1–2 page Word document and asked them to confirm if it was a fair representation and interpretation of their account. Participants were advised that a response was not required or expected if they did not wish to. Of the 22 member check documents sent, 16 responded. All confirmed that they were in agreement with the summary. One requested clarification but was content with the response and agreed that their intended meaning had been understood by the researcher.

To maximise transferability of the findings, purposive sampling was used as the main recruitment technique, which helped maximise data relative to the context. The researcher also used thick description to contextualise the social and cultural relationships of participants, moving beyond mere descriptions of their accounts. This contributes to providing the reader with information so they can judge if the results can be applied to other settings. To achieve dependability, an audit trail was kept by the researcher in the form of a research diary detailing how data were collected and analysed, and how decisions were made throughout the research process.

## 4 Findings

Thirty‐three parents took part in this study (23 mothers and 10 fathers) representing 23 TOPFA experiences. An overview of parents' characteristics is displayed in Table 1. Twenty‐five interviews were carried out, including 19 individual and six dyad. Seven narrative accounts were received: two from participants who were not interviewed and five from individuals who had also been interviewed.

**Table 1:** Parent participant characteristics.

Characteristic	No. of participants
Parents	
Women	23
Men	10
Total	33
Jurisdiction of experience	
Republic of Ireland	22
Northern Ireland	11
Total	33
Legal context of experience (by jurisdiction)	
Republic of Ireland Pre‐Law Change	6
Republic of Ireland Post‐Law Change	16
Northern Ireland Pre‐Law Change	3
Northern Ireland Post‐Law Change	8
Total	33
Gestation at TOPFA	
< 13 weeks	0
13–16 weeks	3
16–20 weeks	5
20–24 weeks	17
24–28 weeks	7
> 28 weeks	1
Total	33
TOPFA location	
Care in Northern Ireland/Republic of Ireland	18
Travelled for TOPFA	15
Total	33
Travelled for TOPFA^a^	
Pre‐law change	9 (3 from NI; 6 from IE)
Post‐law change	6 (all from IE)
Total	15
TOPFA procedure	
Medical induction of labour and vaginal birth	30
Surgical procedure	3
Total	33

a Refers to the 15 participants who travelled for TOPFA.

For all parents in this study, their baby was very much wanted. Although the pregnancy was unplanned for some, no one described it as a crisis pregnancy or considered ending it for any reason before the foetal anomaly diagnosis. The gestation when parents found out about a concern for their unborn baby ranged from 11 to 25 weeks, with the majority finding out at the anomaly scan (*n* = 20 out of 33 parents), carried out at around 20 weeks gestation.

Geographical location, as well as the diagnosis and prognosis of the baby, impacted the trajectory of parents' experience with the period between initial concern and the TFMR procedure ranging from 1 week to 12 weeks among participants (majority 2–4 weeks). For those based in more rural settings, referrals to specialist units impacted timelines between diagnosis and intervention, whereas many of those whose primary care unit had specialist services were able to get quicker investigations and specialist input. In addition, the nature of the anomaly directly impacted whether the parents were able to receive care in Ireland (due to legal restrictions) or if they had to source care elsewhere (England for all parents in this study), which again delayed progress from diagnosis to intervention.

All parents from NI in this study received publicly funded NHS care in NI. Two parents used a private sector provider for antenatal scans, and three paid for a TFMR in England. Although TOP was only decriminalised in NI in 2019, in recognition of the rights of people to access free NHS services within the United Kingdom, from 2017 the UK government provided that TOP care would be paid for women from NI travelling to GB. Before 2017, it was self‐funded. For those participants from IE (*n* = 22), 15 received public maternity care and 7 private care. For all those who travelled to England from IE (*n* = 12), their TFMR care was self‐funded.

This article presents findings related to the impact and the quality of relationships and interactions with HCPs that contributed to parents experiencing care either as enabling and supportive or as unhelpful and difficult. Findings are presented within five themes. The same themes were presented across interviews and narrative accounts, and there were no differences observed between the reported HCP experiences of those in NI and IE. Participant quotes are pseudonymised using a unique code assigned to each individual; PW represents ‘participant woman’, and PP represents ‘participant partner’.

### 4.1 Theme 1: ‘It's All Compassion, It's Just Teaching People That a Loss Is a Loss’—The Importance of Compassionate and Non‐Judgemental Care

The most commonly reported impact on parents' satisfaction of their healthcare experience was whether or not they perceived HCPs' care as compassionate, respectful and empathetic. Words such as ‘caring’, ‘kind’, ‘sensitive’, ‘humane’, ‘attentive’, ‘considerate’, ‘understanding’ and ‘warm’ were used to describe positive experiences.

The midwives were so special, the amount of love they showed us.

(PP03)

Conversely, dissatisfaction with care was due, in most cases, to a perceived lack of compassion shown by HCPs. For some, HCPs’ behaviour was perceived to be brusque or clinically focused. In such instances, when HCPs were seen to display little or no empathy or common courtesy, such as acknowledging the difficult situation parents were facing or introducing themselves, parents described feeling isolated and fearful and used words such as ‘insensitive’ or ‘unresponsive’ to their needs.

You're just treated like a vessel, so the discussions are all about your uterus, but not actually including you in the conversation as a person. I found that very, very tough.

(PW04)

The importance of non‐judgemental care was highlighted by parents who were treated as though their pregnancy was unwanted or unplanned or when HCPs implied their disapproval about parents' choice to have a TOPFA. Failure of HCPs to acknowledge parents’ loss was also experienced as distressing by many.

It was such a relief to be finally treated like I was losing a child and not ending a pregnancy.

(PW08)

Parents highlighted two particular points in their TOPFA experience when compassionate care appeared to be critical: first, when a concern was first raised about the unborn baby's health, and second, during labour.

I will never do them justice with my words, the midwives who held me together [during labour] when I was so afraid.

(PW20)

Partners expressed a desire to be supportive and involved. Those partners who felt enabled to be involved were more positive about their care than those who were not.

I really felt part of it all […] they treated us as a couple.

(PP11)

### 4.2 Theme 2: ‘I Can Handle Knowing What's Going On’—Parents' Value of and Need for Effective Information and Communication

All participants reported that information was important at every stage of their TOPFA journey, from diagnosis through to aftercare. Information was fundamental in helping parents understand the diagnosis and prognosis, what choices they had, what decisions were needed and when. This, in turn, helped them feel a greater sense of agency in a situation that felt beyond their control. Parents also wanted information about the cause of the anomaly and implications for potential future pregnancies. Those who had a more ambiguous prognosis would have liked specialist geneticist input to more fully understand the situation and ensure their decision was fully informed. Parents also appreciated when information was delivered clearly.

They had just been so clear […] I wasn't misled by them in anyway. I asked loads of questions, and they told me as much as they possibly could in the clearest terms that they could […] you know you're just so traumatised and in shock, you really need information given to you clearly, because you have enough to process without trying to filter through what someone is actually saying to you.

(PW05)

Most parents sourced information regarding their baby's diagnosis or prognosis from a variety of sources, mostly online, in addition to that provided by HCPs. Some parents connected with others with lived experience of TOPFA online and reported this as a helpful source of information. Many highlighted how valuable information from others who had experiences a TOPFA would be.

When you're in that tunnel of darkness you need people with experiential knowledge.

(PP06)

Parents who received information about what to expect felt more prepared, especially regarding what to expect in relation to healthcare procedures, options regarding care for the baby, and physical and emotional changes and support. The lack of information regarding what would happen to the baby's body was highlighted as distressing, particularly during the process of a post‐mortem. Parents who were ill‐informed or felt they received inadequate or misinformation from HCPs at any stage of their TOPFA journey reported more distress and dissatisfaction with their healthcare experience. Failure to explain the rationale for actions by HCPs led some parents to interpret behaviours, such as using the staff lift to transfer women between wards, which were possibly well‐intentioned, negatively and to feel judged, ‘I felt like the dirty secret in the hospital’ (PW03).

### 4.3 Theme 3: ‘We Knew That Our Daughter Would be Treated Like a Person’—The Desire for Compassionate Care for Baby and Facilitation of Memory Making

Parents greatly valued when HCPs cared for their baby gently and respectfully. They also wanted their loss to be treated and recognised by HCPs as comparable with other types of pregnancy and baby loss. Parents valued the opportunities provided and facilitated by HCPs to make memories. Tangible mementoes and focused activities with their baby were particularly important. Midwives were the main care provider in this situation and parents spoke fondly of midwives who helped them dress, care for and bond with their baby. When HCPs appeared dismissive, disinterested or did not acknowledge their loss, parents perceived this negatively.

The nurses dressed her in a beautiful little outfit and took hand and footprints and photos for me and my partner, things we will treasure forever.

(PW20)

If parents who have a baby who is born still are offered a box with a blanket and a photograph and a candle or whatever, then the same should be offered to every parent.

(PW03)

### 4.4 Theme 4: ‘We Had an Anchor Around Us, a Consistent Anchor of People’—The Need for Continuity of Care (CoC)

Parents reported increased satisfaction when they experienced CoC during their TOPFA experience. Those who did not receive CoC reported a more disjointed, confusing and overwhelming experience. The lack of CoC was particularly challenging to those parents who travelled to England to access care, where they had to navigate a new health system and build new relationships with HCPs. Parents also found it difficult having to repeat their story during their TOPFA experience and in subsequent pregnancies.

I wish I had someone who would say I'm going to walk with you the whole way […] we had so many different hospitals, so many different doctors, I think just having someone to say this is the next step we're going to go through […] and I'm here to guide you through this, that would have been really helpful to me.

(PW01)

I really appreciated that continuity, because I never had to give my story, everybody knew.

(PW21)

### 4.5 Theme 5: ‘The Irish Should Sort Their Own Out’—Parents' Experiences of Healthcare Relationships During Times of Legislative Change

Fifteen participants had to travel to the United Kingdom for their TOPFA procedure. For nine of these participants (three from NI and six from IE) their TOPFA took place before legislation change. For six participants (all from IE), their TOPFA took place after the legislation change and resulted from the parents’ decision to undergo a TOPFA following an anomaly diagnosis that did not meet the stipulations for TOPFA under Irish law. All parents who travelled reported feelings of distress, fear, anger and abandonment by their HCPs, with most feeling that the experience of having to travel ‘exponentially multiplied’ the ‘nightmare’ (PW06) situation they were living through. Many felt abandoned by the HCPs that they had relied on for support:

It felt like I was in no man's land. Like I don't belong anywhere.

(PW07)

In addition to feelings of shame, isolation and stigma (PW20; PW23), some reported feeling like ‘criminals' (PP06):

I really wish we could have been supported here. It has been the most stressful and heart‐breaking experience of my life […], travelling like this made me feel shame […] I have felt suicidal. If I could have been looked after here maybe I wouldn't feel this way.

(PW23)

The parents from Ireland who had to travel after the legislation change reported shock and disbelief that their HCPs could not support their decision. The legal requirement for two HCPs to agree that a baby will die before or within 28 days of birth was viewed as wrong and dismissed as Ireland ‘paying lip service’ (PP05) to providing access to TOPFA. Some argued that this clause allows people to ‘slip through the cracks […] unless it's cut and dry that the child is not going to survive’ (PW02).

And then my husband was like, ‘do you mean we have to travel?’ And she [HCP] was like, ‘yeah, you'll have to go to London’. And we were just like, what, why? We've voted for this. We repealed the 8^th^ […] we were kind of left stunned.

(PW18)

The fact that some women were unable to receive a TOPFA in Ireland following the change in legislation was considered divisive:

The only women that are traveling are ones that are in the grey area and that is so much more stigmatising for women right now […] I actually feel it was better eight years ago than it is now for women because then everyone had to travel, we were all in literally the same boat. Whereas now we're divided, divided between fatal and not fatal. And it's tough, it's really tough.

(PW07)

Some reported feelings of injustice that there was no help for parents from HCPs in their own country:

With the most wanted and loved unborn little baby who had been dealt the cruellest of hands […] people at their lowest and most vulnerable […] this needs to change, Ireland needs to support its people going through this.

(PP08)

This view was echoed by an English HCP who told a couple that ‘*the Irish should sort out their own*’ (PP02).

Some participants commented on how the legislative changes appeared to have heightened fear of prosecution for HCPs. They felt it created a culture of defensive and unhelpful practice because HCPs ‘would be worried about getting sued for making recommendations of what to do and where to go’ (PP05). One woman was asked to consent to a post‐mortem, which she believed was partly so that the consultant could ‘confirm (his) diagnosis was a hundred percent accurate’ (PW20).

Another couple said they felt ‘abandoned’ by their doctor in Ireland who had advised them to travel to England to receive a foetcide procedure and then return home and present themselves to the local hospital with ‘reduced fetal movement’ for labour and birth care, advising them not to tell the staff what they had done as ‘some midwives would not treat them if they knew’ (PW02; PP02). The couple travelled to England but were unable to have the procedure as their doctor in Ireland denied knowledge of the couple and would not confirm that follow‐up care would be provided in Ireland when asked by the doctor in England.

Conscientious objection of HCPs to TOPFA was identified as an issue by several participants with the view that it made, ‘the situation ten times worse and it's making those couples feel like criminals’ (PW04). There was consensus that HCPs who have a conscientious objection should not be involved in the care of families going through a TOPFA:

I know we all have our thoughts about things but if you are really that way inclined, you're in the wrong job.

(PW04)

I think that for any service that it would be very important that only people who are supportive would be involved in any team because it's subtle, but it becomes very obvious very quickly who's judgmental and who's not.

(PW08)

Parents said that they felt like ‘criminals’ when things happened in a clandestine manner magnifying their sense of isolation, fear and shame:

The whole situation here, it's all clandestine, it's all cloak and dagger stuff. They need to be more transparent.

(PW04)

Of the parents who received their care at home following legislation change, most felt that HCPs were not prepared:

I just feel we had all of Ireland's inexperience to deal with. It was the first time for everyone.

(PW03)

## 5 Discussion

This study increases understanding of the important role HCPs play in parents’ TOPFA experience and highlights the impact they can have on how parents experience their care. These findings are particularly important to inform TOPFA‐related healthcare service changes and decision‐making on the Island of Ireland (and other similar jurisdictions) during a time of legislative change and health service reform.

### 5.1 Person‐Centred Care

Putting patients at the centre of care and providing respectful, compassionate, empowering and individualised care is accepted as good practice for HCPs and healthcare systems in NI and IE [42, 43, 44, 45, 46, 47, 48, 49]. This approach, referred to widely as person‐centred care, has received much attention in policy, education and practice [43, 50, 51, 52]; however, there is no single agreed definition [53]. Promoting compassion and kindness in health systems based on relationships and respect is also not new. The findings of this study, however, suggests this has not yet been fully realised in practice relating to TOPFA [54, 55]. Although some UK studies have reported congruence between HCP conceptualisations and understandings of parents’ experiences [56], this study suggests that, in the context of Ireland, parents do not always experience this understanding and congruence in practice. A partial explanation for this may be offered by the upheaval and uncertainty caused by the context of legislative change, as well as the challenges of provision for HCPs in other jurisdictions [57, 58]; however, further research comparing HCP and parent understandings and experience in this context is warranted [59].

### 5.2 Effective Communication

Parents valued HCPs who they felt they could trust and who communicated effectively and honestly with them, particularly when sharing difficult news or complex information. Communication and information are recognised elements of effective person‐centred care [60]. A blend of clinical competence and compassionate care must coexist in practice for person‐centred care to be realised [61]. In this study, there was a stronger focus by parents on compassion, with little focus on how clinically competent HCPs were, suggesting parents implicitly expected HCPs to be clinically competent and behave professionally. That being said, communication from HCPs with parents who do not fall within the remit of qualifying for a TOPFA in their home country, was highlighted as a significantly negative experience for some participants. Communication in such circumstances requires an especially sensitive and compassionate approach, and this study highlights some of the challenges that HCPs face in relation to this. Further guidance and training for HCPs, as well as a clear pathway of care for such parents, particularly when they decide to travel abroad for TOPFA, is urgently required to safeguard both HCPs and parents [62, 63, 64].

Parents in this study had different experiences on how well they were communicated with. This could be for a variety of factors from personality, self‐protection, lack of experience or skill, burnout, insufficient or poor training or not being a priority in professional revalidation or performance management [65]. A recent study of the use of self‐directed learning in pregnancy loss for student nurses showed statistically significant effects on perceived knowledge, confidence and skills in caring for families experiencing pregnancy loss [66]. A systematic review on educational interventions for nursing students to develop communication skills reported an increase in skills following most interventions, especially simulation [67]. However, differences in study design and a lack of long‐term evaluation of impact make it difficult to determine effectiveness over time. The need for a focus on training and refresher training in communications skills for HCPs is supported by this study's findings. Consideration should be given to simulation experience design [68] and a longitudinal study design to assess the long‐term improvements in communication skills alongside observational studies to assess performance in clinical practice [69].

### 5.3 Compassion, Kindness and Non‐Judgmental Care

Compassionate care is supported in principle within healthcare systems and by health professionals; however, this study supports the findings of others, which suggest that compassionate care is not consistently experienced by patients [70, 71, 72, 73, 74]. Universally, parents involved in this study wanted to be treated with dignity, kindness and compassion and to feel they could trust HCPs to support and care for them. HCPs who were compassionate were viewed positively by parents who felt they were shown respect and their decision to have a TOPFA was not being judged. Those who felt they were not cared for compassionately by HCPs remembered the negative impact of the HCPs' behaviours or words and recalled the shock, distress and anger they had felt at the time.

Having a TOPFA is a stressful and distressing experience. Stress and distress can distort judgement, communication and outcomes [75]. HCPs should understand how these feelings can heighten parents’ sensitivity to any suggestion of judgement from others, whether in word or deed. A trauma‐informed approach to care may be useful to consider for a TOPFA service and could help ensure parents feel treated with dignity, respect and compassion [76]. Literature exploring trauma victims' interactions with the police service could be beneficial in helping HCPs understand the intensity of the experience from the parent's perspective and how behaviours, actions and words can impact on how safe an individual feels during or following a traumatic event [77, 78].

### 5.4 Memory Making

Historically within the healthcare system, parents who lost a baby before, during or at birth were not offered or given the opportunity to see or hold their baby [79, 80]. A shift in the late 1970s and early 1980s saw practice change [81, 82], with the contemporary practice now promoting parental contact and memory‐making in cases of neonatal death [73, 74, 75, 76, 77, 78, 79, 80, 81, 82, 83, 84, 85]. However, seeing and holding the baby may not be desired or beneficial for everyone, further promoting the importance of choice [86, 87]. Reflective of other baby loss literature, parents appreciated midwives' presence following the birth of baby and their support in helping make memories [88, 89, 90]. Parents in this study also appreciated how the midwife handled their baby gently, used their name and showed care and compassion, echoed in other literature [91].

### 5.5 CoC

Current policy in the United Kingdom, NI and IE promotes CoC models within maternity care [92, 93, 94, 95]. A number of quantitative and qualitative studies in maternity services report improved outcomes for both mother and baby and increased satisfaction linked to CoC [96, 97, 98, 99, 100, 101]. Consistent with this research, parents in this study reported increased satisfaction when they experienced CoC during their TOPFA experience. Many parents suggested that a named HCP to provide guidance, advocate for their needs and wishes and oversee their care pathway would be helpful. Consideration should be given by service providers to establish dedicated posts to help parents navigate their TOPFA journey. This could have the added advantage of HCPs being able to build up experience and expertise in this area of practice. As noted, special consideration also needs to be given to those who decide to travel outside their home jurisdiction for TOPFA in relation to after care provision. The terrain for understanding what is acceptable within the confines of current legislation remains grey, but few would argue the necessity for providing appropriate healthcare to those who need it. Guidelines for best practices are urgently needed.

## 6 Strengths and Limitations

**Table:** 

This is the first study in IE and NI to explore the holistic healthcare experience of parents who have TOPFA pre‐ and post‐legislative changes offering a timely opportunity to contribute towards shaping and improving services in these and similar contexts. The study sample includes both women and men and has a balanced geographical spread from IE and NI, providing a breadth of parental experiences. The involvement of an AG throughout the research process was also a strength. The group provided advice, assurance and quality control to ensure that the language and terminology in participant‐facing documents were appropriate and sensitive. This study was exploratory in nature and therefore did not attempt to make generalisations across demographically diverse locations or populations. However, findings are likely to be transferrable in other similar contexts and settings. We did not collect information on the type of diagnosis, religious or ethnic background, or length of time between TOPFA and the interview. Consideration of these variables may have allowed examination of variability in experiences and warrant further research.

## 7 Conclusion

This research highlights the important role HCPs play in contributing to how parents cope through a TOPFA. Parents who had a positive relationship with an HCP, in which information was communicated effectively, and the care provided compassionate and non‐judgemental, appeared to feel supported through their TOPFA experience. Conversely, any interaction with an HCP that was perceived to be negative resulted in heightened parents' distress and increased their feelings of isolation and withdrawal.

## Author Contributions

**Suzanne Heaney:** conceptualisation, investigation, writing–original draft, methodology, project administration, formal analysis. **Mark Tomlinson:** supervision, conceptualisation. **Áine Aventin:** writing–review and editing, conceptualisation, funding acquisition, methodology, project administration, supervision.

## Conflicts of Interest

The authors declare no conflicts of interest.

## Supporting information

Supporting information.

## Data Availability

The data that support the findings of this study are available on request from the corresponding author (Á.A.). The data are not publicly available due to restrictions on information that could compromise the privacy of research participants.
