# Supplementary material for: Perceived Impact of Healthcare Relationships and Interactions on Parental Experiences of Prenatal Diagnosis and Termination of Pregnancy for Foetal Anomaly on the Island of Ireland
Source: Health Expect. 2024 Oct 20;27(5):e70068. doi: 10.1111/hex.70068 (PMC11491545; doi:10.1111/hex.70068)
Supplement: Supplementary file 1 — Supporting information. [file HEX-27-e70068-s001.docx]

**Supplementary Material**


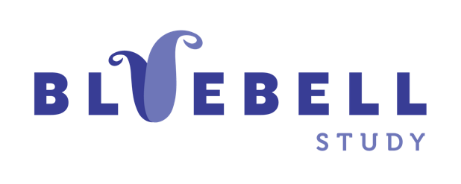

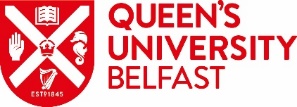


**THE BLUEBELL STUDY: AN EXPLORATION OF THE HEALTHCARE EXPERIENCES AND NEEDS OF PARENTS WHO TERMINATE A PREGNANCY IN THE CASE OF A FETAL ANOMALY DIAGNOSIS ON THE ISLAND OF IRELAND**

**Interview Guide and Prompts**

**PARENTS**

| **TOPIC** | **PROMPTS** |
| --- | --- |
| **Introduction** | - Overview of study - Available Support - Thank you for time and sharing story - Acknowledge importance of language – ascertain what they would like to be called, what they would like their baby to be called, whether they would prefer the term ‘abortion’ or ‘termination of pregnancy’ to be used - Confirm consent - Confirm they are happy to proceed |
| **Background** | We would like to ask you some questions about your background, to help us get an insight in to our participant group. Please be assured none of this information will be identified to you.  It will remain anonymous and confidential.   - Current age - Age at time of TOPFA - Where were you living - Were you working - Religious background |
| **Pregnancy - Diagnosis** | - Pregnancy number - Feelings about the pregnancy - Experience of early pregnancy |
| **Diagnosis** | - Gestation at diagnosis - Where did you find out? - Who delivered the news to you? - Can you tell me a bit about the anomaly/condition? - Information or options given - Feelings about the discussion of options |
| **Decision** | - Information given/found and sources - Support to make the decision - Who did you talk to? - Belief/perception about abortion |
| **Experience of TOPFA** | - Procedure - Travel? Where to? - Costs - Family knowledge and support - Experience of health professionals - Offered any bereavement support? - Were you offered post-mortem? Feelings on this. - What were the options for your baby’s remains? And what did you choose to do? |
| **Post abortion care** | - Physical needs/follow up - Support services |
| **Experience of Healthcare Staff** | - Anything done well or not well - Did you find staff supportive? |
| **Communication** | - Experiences about communication and language - Did you feel you had to repeat your story? - Did you feel you had choices? |
| **TOPFA - now** | - Experience - Another pregnancy? - feelings? care? |
| **Law change** | - Thoughts on the law change in NI/ROI |
| **Future** | - What would have helped? - What do you think parents in this situation need? |
|  | - Anything further you would like to add or ask about? |
